# Tuberculin in Phthisis

**Published:** 1913-01

**Authors:** 


TUBERCULIN IN PHTHISIS. John Guy, *Glasgow Med. Jour.*, No. 3, 1912, believes that the opsonic index is useless, that tuberculin can be administered with comparative safety if the temperature is watched, and that there is no general clinical evidence as to the superiority of any particular form of tuberculin.

